# Associations between road traffic noise exposure at home and school and ADHD in school-aged children: the TRAILS study

**DOI:** 10.1007/s00787-020-01521-8

**Published:** 2020-04-03

**Authors:** W. L. Zijlema, Y. de Kluizenaar, I. van Kamp, C. A. Hartman

**Affiliations:** 1 Barcelona Institute for Global Health (ISGlobal), Barcelona Biomedical Research Park (PRBB), C/Doctor Aiguader 88, 08003 Barcelona, Spain; 2 Universitat Pompeu Fabra (UPF), Doctor Aiguader 88, 08003 Barcelona, Spain; 3 CIBER Epidemiología y Salud Pública (CIBERESP), Melchor Fernández Almagro, 3-5, 28029 Madrid, Spain; 4 TNO, Stieltjesweg 1, 2628 CK Delft, The Netherlands; 5 RIVM, Antonie van Leeuwenhoeklaan 9, 3721 MA Bilthoven, The Netherlands; 6 Department of Psychiatry, University of Groningen, University Medical Center Groningen, Groningen, The Netherlands

**Keywords:** Attention deficit/hyperactivity disorder, Road traffic noise, Adolescents, Child health, Cross-sectional study

## Abstract

Environmental noise may play a role in the manifestation and severity of attention deficit/hyperactivity disorder (ADHD) symptoms, but evidence is limited. We investigated the cross-sectional associations between residential and school road traffic noise exposure and ADHD symptoms and diagnosis. The sample included *n* = 1710, 10–12-year-old children from the TRAILS study in The Netherlands. ADHD symptoms were measured using a DSM-IV based subscale from the Child Behavior Checklist. Children with diagnosed ADHD originated from the clinic-referred cohort. Road traffic noise (*L*_den_) was estimated at the residence and school level, by model calculation. Risk ratios for ADHD symptoms and ADHD diagnoses, and regression coefficients for symptom severity were estimated separately and simultaneously for residential and school road traffic noise. Adjusted multinomial models with residential road traffic noise showed that residential noise was not associated with ADHD symptoms, but was associated with lower risks for ADHD diagnosis (RR = 0.93; 95% CI 0.89, 0.97). Similar associations were observed for models including school road traffic noise and models including both exposures. No clear exposure response relationship was observed for associations between residential or school noise and ADHD symptom severity. We found no evidence for a harmful association between road traffic noise and ADHD. Associations between noise and lower risks for ADHD were observed only in referred cases with a confirmed ADHD diagnosis and may be due to residual confounding or selection bias. Future studies should focus on residential and school noise exposure, and study associations with ADHD symptoms and diagnosis over time.

**Electronic supplementary material:**

The online version of this article (10.1007/s00787-020-01521-8) contains supplementary material, which is available to authorized users.

## Introduction

Attention deficit/hyperactivity disorder (ADHD) is a neurodevelopmental disorder with symptoms continuing into adulthood [1, 2]. ADHD is characterized by a significant impairment in the functioning of a child due to elevated inattention and distractibility on one hand and excessive hyperactivity or impulsivity on the other. ADHD is among the most common disorders among children with a prevalence of 6–7% [3].

Although the exact causes of the disorder have not yet been identified, it is evident that both biological as well as environmental factors are accountable for the manifestation of the disorder. Research on biological factors primarily involves genetics and brain structure and function [4, 5]. ADHD occurs more often in boys than in girls and is associated with lower socioeconomic status (SES) [1]. Identified risk factors for the development of ADHD include pregnancy and delivery complications, maternal smoking and alcohol use during pregnancy, and an unstructured home environment (e.g., frequent family conflict, maternal psychopathology, paternal criminality, and negative and less rewarding attitude towards the child) [1, 6].

Studies have shown associations between environmental noise and ADHD symptoms. A large cohort study performed in 46,940 children from Denmark found that a 10 decibel increase in road traffic noise exposure from birth to age 7 was associated with a 9% increase in borderline and abnormal hyperactivity/inattention subscale scores [7]. Similar conclusions were taken from a relatively small cross-sectional German study (*n* = 872) that reported that higher road traffic noise levels were associated with more hyperactivity/inattention symptoms [8]. Road traffic noise at age 8 and during the last 5 years was associated with inattention at age 8 in a sample (*n* = 1384) from Oslo, Norway [9]. Most of the previous studies focused on residential road traffic noise exposure, but a cross-sectional study in Barcelona in children aged 7–11 years (*n* = 2897) focused on traffic noise at schools, and found that noise exposure at the school was associated with ADHD symptoms [10]. Furthermore, exposure to aircraft noise at schools has also been associated with hyperactivity symptoms [11, 12].

Other previous studies focused on adverse effects of noise on children’s cognition. Particularly, tasks relying on sustained attention (e.g., reading skills and working memory) were found to deteriorate as a result of exposure to traffic noise [11, 13–17], but more robust evidence is needed [18]. Typical for ADHD is a lower noise tolerance level, as children with ADHD are more easily distracted by noise in the classroom [19], and experience a lower level of comfort and tolerance for spoken language compared to children without ADHD [20]. Furthermore, not only the noise itself, but also the subjective evaluation of noise can be a stressor resulting in adverse health effects [21, 22]. Noise sensitive people feel more threatened by noise, react more to noise, and adapt more slowly to noise compared to people who are less noise sensitive. It is thought that noise sensitivity is linked with negative affectivity and physiological arousal to noise, and it might, therefore, be linked to ADHD symptoms [23].

Environmental noise may play a role in the manifestation and severity of ADHD symptoms, but evidence is limited. Most of the previous research solely assessed ADHD symptoms and not clinical diagnosis. They further focused on residential road traffic noise alone, while children spend a large part of their day at school. To address these gaps, we investigated the associations between residential and school road traffic noise exposure (separately and simultaneously) and ADHD symptoms and diagnosis.

## Methods

### Sample

Participants were a subsample from the population (*n* = 2230) and clinic-referred (*n* = 543) cohorts of TRAILS (Tracking Adolescents’ Individual Lives Survey). Details about this cohort and the recruitment of participants have been published previously [24, 25]. In brief, TRAILS is a prospective study of Dutch adolescents with bi- and triennial measurements since age 11 [25]. Our analysis was cross-sectional and focused on data from the first measurement wave. Children in the population sample were recruited through schools in urban and rural areas in the North of The Netherlands. Children in the clinic-referred cohort had been referred to the Groningen University Child and Adolescent Psychiatric Outpatient Clinic at any point in their life (20.8% ≤ 5 years, 66.1% 6–9 years, and 13.1% 10–12 years) for consultation or treatment.

**Fig. 1:**
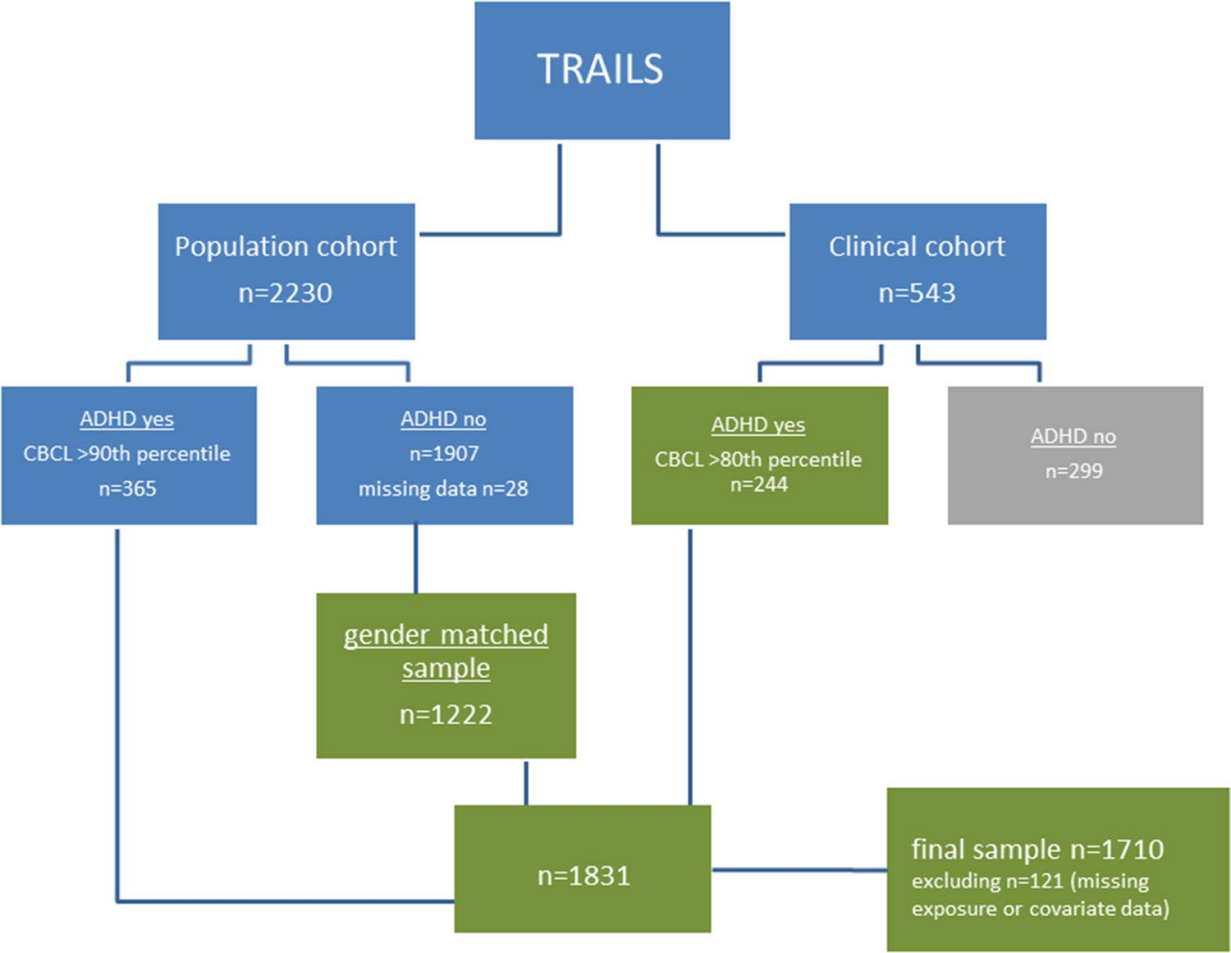
Selection of study sample from TRAILS population and clinical cohort

For the present study, we sampled children with a lifetime diagnosis of ADHD and currently elevated ADHD symptoms (i.e., not in remission; *n* = 244) at age 11 from the TRAILS clinical cohort. In the population cohort, we identified a subsample who screened positive for possible ADHD, and randomly drew a gender-matched reference sample from those who screened negative (male–female ratio 2:1; this ratio coincided for the diagnosed and screen-positive subsamples). See Fig. 1 and further description of this selection, as based on the ADHD measurement instruments, below. This sampling strategy allowed us to compare manifestations of ADHD symptoms in a clinical and population sample.

The study was approved by the Dutch Central Committee on Research Involving Human Subjects (CCMO). The study was carried out in accordance to the Declaration of Helsinki and all measurements were carried out with their adequate understanding and written consent.

### Attention deficit/hyperactivity disorder

In the TRAILS clinical cohort, information on the presence or absence of ADHD was assessed with the Diagnostic Interview Schedule for Children (DISC-IV parent version) [26]. In addition, parental and teacher ratings were used from the DSM-based ADHD scale of the Child Behavior Checklist (CBCL) and a short version of the Teacher Rating Form (TRF) [27, 28] to ensure the presence of current symptoms rather than full remission (as based on the 80th percentile of scores on either parent or teacher scale in the normative general population cohort). This subscale consists of seven items (attention problems: fails to finish; cannot concentrate, inattentive; hyperactive-impulsive problems: cannot sit still; impulsive; talks too much; loud), which were averaged. The CBCL and TRF are questionnaires for assessing behavioral and emotional problems in the past 6 months in 4–18 years old. The response format is 0 = not true, 1 = somewhat true, and 2 = very true or often true [27].

In the TRAILS population cohort, the diagnostic interview DISC was not administered. Therefore, we identified children who had high scores of ADHD symptoms, based on a score on the ADHD subscale of the CBCL or short version of the TRF above the 90th percentile in our normative general population cohort. Average symptom levels in the diagnosed ADHD group from the clinical sample were similar to those in the ADHD screen-positive group from the population sample. From the population cohort, a gender-matched sample (i.e., with the same male/female ratio as the diagnosed and screen-positive groups) of *n* = 1222 was selected and added to the population cohort study sample. Finally, 121 individuals were excluded due to missing data on traffic noise exposure or covariates yielding a final sample of *n* = 1710 (Fig. 1; 229 confirmed children with ADHD and 341 who screened positive for ADHD symptoms from the clinical and population cohort, respectively).

### Road traffic noise exposure

Road traffic noise was calculated at the residence and school levels. Calculations were done at the most exposed facade of the building using the STAMINA (Standard Model Instrumentation for Noise Assessments) in accordance with the requirements of the European Environmental Noise Directive (END). The road traffic noise model was based on 2011 data, and included information on traffic intensity, speed, composition, type of road surface, building data, and surface type. We used the EU standard noise metric *L*_den_. *L*_den_ is the average 24 h sound level, with a 10 dB penalty added to the levels between 23.00 and 07.00 h and a 5 dB penalty added to the levels between 19.00 and 23.00 h to reflect people’s extra sensitivity to noise during the night and the evening. Road traffic noise exposure was calculated at 4 m above the ground of the dwelling facade of the exposed subject [29].

### Covariates

The covariates were chosen a priori based on the previous studies [7–10]. Data on age, sex, the number of parents in the household, whether parents were foreign-born, maternal smoking and alcohol use during pregnancy, problems during pregnancy and child delivery, mothers age at child birth, pregnancy duration (weeks), birth weight, and household size (number of children and adults excluding parents living at the residence; as a measure of crowding) were obtained from the parent questionnaire. Parental education, parental job title, and parental income were obtained with questionnaires, categorized and ordered, and finally combined by averaging the indicators after standardization into a score reflecting parental socioeconomic status (SES) [30]. Children’s use of psychostimulant medication was likewise reported by their parents. Lifetime parental psychopathology with respect to depression, anxiety, substance dependence, and persistent antisocial behavior was assessed with an interview. For each spectrum, each parent was assigned to one of the following categories: 0 = (probably) never had an episode, 1 = (probably) yes, or 2 = yes and treatment and/or medication. Two *z*-scores were computed reflecting parental internalizing problems (anxiety and depression) and parental externalizing problems (antisocial behavior/substance abuse) [31]. Finally, data on quality of the residential environment and noise sensitivity were only available for children in the population cohort. Quality of the residential environment was assessed by the interviewer with the following items:

- Unpleasant indoor environment (tobacco smoke, noise, little natural light, and poor insulation).
- Dirty indoor environment (dust, dirty surfaces, messiness).
- Comfortable home (large living room and kitchen, pleasant garden).
- Spacious, pleasant outdoor environment (family friendly neighborhood, playgrounds, parks, and low-density traffic).

All items were answered on a 5-point scale ranging from strongly disagree to strongly agree. The four questions were normalized and scores were summed into a quality score ranging from 4 to 20, with higher scores indicating higher quality of the residential environment.

For noise sensitivity, children answered the following question: “I notice sounds around me before other people do (ticking clocks, dripping taps)”, on a 5-point scale ranging from (1) “almost never true”, to (5) “almost always true”.

### Statistical analysis

Descriptive statistics were used to characterize the study population, and were calculated for the pooled sample and for those without ADHD, those with ADHD symptoms, and those with an ADHD diagnosis as described above. Equivalence tests (ANOVA, Chi-square, and Kruskal–Wallis tests) and were used to test for differences between groups. Risk ratios and regression coefficients for ADHD were estimated for residential *L*_den_ and school *L*_den_ as follows:

1. Multinomial regression analyses were used to estimate associations between residential and school road traffic noise (*L*_den_) and ADHD with multinomial regression analyses. Participants were classified into three groups: no ADHD symptoms (reference group); ADHD symptoms in population cohort; and ADHD diagnosis in clinical cohort. Models were stepwise adjusted for: (1) age and sex; (2) parental SES, single parenthood, parents born outside The Netherlands; (3) maternal smoking during pregnancy, maternal alcohol use during pregnancy, problems during pregnancy or delivery; and (4, main model) parental internalizing and externalizing problems. Associations were estimated separately for residential and school road traffic noise, but also simultaneously including noise estimated for the residence and school.
2. Linear regression analyses were undertaken with ADHD symptom severity as outcome. This was done (i) in the total sample; (ii) in a sample with those with ADHD symptoms and ADHD diagnosis; and (iii) in only those with an ADHD diagnosis. We furthermore performed linear regression analyses with symptom severity scores for two ADHD subscales: attention and hyperactivity/impulsivity in these groups. In addition to analyses with continuous *L*_den_, we also estimated associations with *L*_den_ categories < 50 dBA; 50–60 dBA; and > 60 dBA. These models were adjusted for the same set of covariates as specified above (main model).
3. Multinomial regression analyses (see above at 1.) stratified for sex, parental SES (stratification based on median), and parental internalizing and externalizing problems (stratification based on median), adjusted for the same set of covariates as specified above (main model, excluding the moderators) were undertaken to evaluate sex, SES, and parental psychopathology differences in the relationship between *L*_den_ and ADHD symptoms and diagnosis.

For the first set of analyses (see above at 1.), we performed sensitivity analyses by applying a more stringent cut-off for ADHD symptoms in the population cohort and defined an ADHD score above the 95th percentile of the CBCL or the short form of the TRF as indicative of the presence of ADHD problems [27]. We further adjusted the main model (see above at 1.) for pregnancy duration, birth weight, and household crowding to assess sensitivity to these factors. Finally, since data on noise sensitivity and residential environment quality were available in the TRAILS population sample only, we performed sensitivity analyses with these variables that were restricted to the population sample. We assessed associations between exposure to road traffic noise at home and school, and ADHD symptoms stratified by noise sensitivity (low: 1–3 and high: 4–5); and associations between noise sensitivity itself and ADHD symptoms in the TRAILS population cohort. We further adjusted the main model for residential environment quality to assess potential confounding. Analyses restricted to the population sample were logistic regression analyses adjusted for the same covariates as in the main model.

Data were analyzed for participants from whom we had complete data (*n* = 1710 for residential and *n* = 1538 for school *L*_den_ analyses). Effect estimates are presented as risk ratios (RR; ADHD symptoms and ADHD diagnosis) or regression coefficients (*β*; symptom severity), or odds ratios (OR; ADHD symptoms) with 95% confidence intervals (CI), per 1 dB(A) or categories of *L*_den_. Associations were considered statistically significant if the 95% confidence intervals did not include one (RR) or zero (*β*). Analyses were performed using the statistical software package STATA version 14.2 [32].

## Results

Population characteristics of the total sample and by ADHD status are presented in Table 1. Overall, mean residential road traffic noise exposure was 53.0 (range 32.3–72.4) dB(A) and 53.2 (range 36–72.4) dB(A) at school. Children who screened positive for ADHD symptoms from the population cohort had parents with lower SES, had mothers who were younger during childbirth, and had more often parents that smoked during pregnancy compared to children without ADHD from the population cohort and those with ADHD diagnosis from the clinical cohort. Children with an ADHD diagnosis from the clinical cohort had less often a parent that was born outside The Netherlands and had more often parents with a history of psychopathology compared to the other children. Average residential and school road traffic noise was lower for children with ADHD in the clinical cohort, compared to children from the population cohort (regardless of ADHD symptoms). Residential road traffic noise and school road traffic noise were not correlated (*r* = 0.012), and neither were noise and parental SES (residential road traffic noise *r* = − 0.003; school road traffic noise *r* = 0.081).

**Table 1:** Characteristics of the TRAILS study population by ADHD status (*n* = 1710)

	Total *n* = 1710	No ADHD *n* = 1140	ADHD symptoms *n* = 341	ADHD diagnosis *n* = 229	*p* value
Age, mean (SD)	10.6 (0.63)	10.6 (0.63)	10.6 (0.67)	10.6 (0.54)	0.653
Sex, *n* (%)					0.911
Males	1167 (68.3)	775 (68.0)	233 (68.3)	159 (69.4)	
Females	543 (31.8)	365 (32.0)	108 (31.7)	70 (30.6)	
Home *L*_den_, mean decibel (A) (SD)	53.0 (5.03)	53.2 (4.94)	53.1 (4.92)	51.8 (5.48)	< 0.001
School *L*_den_, mean decibel (A) (SD)	53.2 (5.52)	53.5 (5.63)	53.2 (5.32)	51.8 (5.04)	< 0.001
Parental SES, mean *z*-score (SD)	− 0.070 (0.79)	0.024 (0.80)	− 0.35 (0.75)	− 0.12 (0.69)	< 0.001
Single parent status, *n* (%)					< 0.001
One parent	257 (15.0)	139 (12.2)	69 (20.2)	49 (21.4)	
Two parents	1453 (85.0)	1001 (87.8)	272 (79.8)	180 (78.6)	
Household size (excl. parents), mean (SD)	2.52 (1.12)	2.54 (1.04)	2.49 (1.26)	2.50 (1.27)	0.719
Country of birth parents, *n* (%)					0.001
Parents born in NL	1559 (91.2)	1027 (90.1)	308 (90.3)	224 (97.8)	
At least one parent born outside NL	151 (8.83)	113 (9.91)	33 (9.68)	5 (2.18)	
Mothers age at birth child, mean (SD)	29.3 (4.52)	29.6 (4.42)	28.1 (4.65)	29.7 (4.60)	< 0.001
Smoking during pregnancy, *n* (%)					< 0.001
Yes	514 (30.1)	308 (27.0)	134 (39.3)	72 (31.4)	
No	1196 (69.9)	832 (73.0)	207 (60.7)	157 (68.6)	
Alcohol during pregnancy, *n* (%)					0.323
Yes	302 (17.7)	197 (17.3)	69 (20.2)	36 (15.7)	
No	1408 (82.3)	943 (82.7)	272 (79.8)	193 (84.3)	
Pregnancy duration, mean weeks (SD)	39.8 (2.13)	39.8 (2.01)	39.7 (2.17)	39.7 (2.65)	0.685
Birth weight, mean (SD)	6.84 (1.49)	6.84 (1.18)	6.76 (1.41)	6.99 (2.57)	0.192
Problems during pregnancy/delivery, *n* (%)					< 0.001
Not at all	783 (45.8)	574 (50.4)	131 (38.4)	78 (34.1)	
A little	827 (48.4)	563 (49.4)	209 (61.3)	55 (24.0)	
Quite some or a lot	100 (5.85)	3 (0.26)	1 (0.29)	96 (41.9)	
Parental internalizing problems, median score (IQR)	0.59 (0.81)	0.50 (0.76)	0.69 (0.85)	0.89 (0.87)	< 0.001
Parental externalizing problems, median score (IQR)	0.16 (0.45)	0.12 (0.37)	0.22 (0.54)	0.27 (0.60)	< 0.001
Quality of the residential environment, median score (IQR)	18 (5)	18 (4)	17 (6)	n.a	< 0.001
Noise sensitivity, *n* (%)					0.386
Almost never true	370 (27.1)	291 (78.7)	79 (21.4)	n.a	
Usually not true	224 (16.4)	180 (80.4)	44 (19.6)	n.a	
Sometimes true	457 (33.5)	346 (75.7)	111 (24.3)	n.a	
Usually true	172 (12.6)	133 (77.3)	39 (22.7)	n.a	
Almost always true	141 (10.3)	102 (72.3)	39 (27.7)	n.a	
ADHD score, item average, mean (SD)	0.76 (0.55)	0.43 (0.31)	1.29 (0.36)	1.46 (0.35)	< 0.001
Inattention, item average, mean (SD)	0.85 (0.63)	0.52 (0.44)	1.43 (0.43)	1.59 (0.41)	< 0.001
Hyperactivity/Impulsive, item average, mean (SD)	0.67 (0.58)	0.37 (0.34)	1.19 (0.46)	1.36 (0.47)	< 0.001
Medication use	157 (9.18)	5 (0.44)	29 (8.50)	123 (53.7)	< 0.001

### Road traffic noise, ADHD symptoms, and ADHD diagnosis

Multinomial regression analyses showed that higher residential road traffic noise levels were associated with lower risks for ADHD diagnosis, but not for ADHD symptoms. The association between noise and ADHD diagnosis remained statistically significant in the fully adjusted model (RR 0.929, 95% CI 0.893, 0.965; Table 2). Analyses with school road traffic noise and ADHD also showed that higher noise levels were related to lower risks for ADHD diagnosis (adjusted RR 0.945, 95% CI 0.910, 0.981), but not with ADHD symptoms. Results did not change when associations between residential and school road traffic noise and ADHD were analyzed simultaneously (Table 2).

**Table 2:** Associations between exposure to road traffic noise at home (*n* = 1710) and school (*n* = 1538) and ADHD in the TRAILS cohort

		*N* cases	Risk ratio for ADHD (95% confidence interval)			
			m1	m2	m3	m4
*L*_den_ home (per 1 dBA)	ADHD symptoms	341	0.995 (0.971, 1.019)	0.995 (0.970, 1.020)	0.994 (0.969, 1.020)	0.994 (0.969, 1.019)
	ADHD diagnosis	229	0.942 (0.913, 0.971)*	0.941 (0.912, 0.970)*	0.930 (0.895, 0.967)*	0.929 (0.893, 0.965)*
*L*_den_ school (per 1 dBA)	ADHD symptoms	293	0.988 (0.965, 1.012)	0.996 (0.971, 1.020)	0.996 (0.972, 1.021)	0.997 (0.972, 1.022)
	ADHD diagnosis	222	0.940 (0.913, 0.967)*	0.945 (0.917, 0.973)*	0.943 (0.909, 0.979)*	0.945 (0.910, 0.981)*
*L*_den_ home (per 1 dBA)	ADHD symptoms	293	0.991 (0.966, 1.018)	0.991 (0.965, 1.018)	0.991 (0.964, 1.019)	0.990 (0.963, 1.017)
*L*_den_ school (per 1 dBA)			0.988 (0.964, 1.012)	0.996 (0.971, 1.020)	0.996 (0.972, 1.021)	0.997 (0.973, 1.022)
*L*_den_ home (per 1 dBA)	ADHD diagnosis	222	0.941 (0.912, 0.971)*	0.941 (0.912, 0.971)*	0.925 (0.888, 0.963)*	0.921 (0.885, 0.959)*
*L*_den_ school (per 1 dBA)			0.941 (0.914, 0.968)*	0.946 (0.919, 0.974)*	0.946 (0.912, 0.982)*	0.948 (0.914, 0.984)*

*M1* adjusted for sex, age; *M2* M1 + parental SES, number of parents, ethnicity; *M3*: M2 + perinatal circumstances and complications; *M4*: M3 + parental externalizing and internalizing problems. Based on multinomial regression analysis with screen-negative for ADHD as reference group (*n* = 1140/1023)

*p < 0.05

### Road traffic noise and ADHD symptom severity

We observed no associations between residential road traffic noise and ADHD symptom severity in the pooled sample, in the sample with ADHD symptoms and diagnosis, nor in those with an ADHD diagnosis. Coefficients were generally close to zero and were not statistically significant (Table 3a). School road traffic noise > 60 dBA (vs. < 50 dBA) was associated with − 0.113 (95% CI − 0.209, − 0.018) lower scores for ADHD symptom severity in the pooled sample, but no associations were observed for continuous *L*_den_ and in the subsamples (Table 3a).

**Table 3:** Associations between exposure to road traffic noise at home and school, and ADHD symptom severity in TRAILS population and clinical cohort, in all ADHD cases, and in ADHD cases from clinical cohort

A	Regression coefficient for ADHD severity (95% confidence interval)		
	Pooled	ADHD symptoms and diagnosis	ADHD diagnosis
*L*_den_ home (continuous)	*n* = 1627 − 0.005 (− 0.009, 0.000)	*n* = 552 0.001 (− 0.005, 0.007)	*n* = 225 0.008 (− 0.001, 0.016)
* L*_den_ home < 50 dBA	*n* = 283 Reference	*n* = 170 Reference	*n* = 87 Reference
50–60 dBA	*n* = 1022 − 0.039 (− 0.095, 0.018)	*n* = 344 − 0.023 (− 0.091, 0.045)	*n* = 125 0.003 (− 0.096, 0.102)
> 60 dBA	*n* = 152 − 0.080 (− 0.173, 0.013)	*n* = 38 − 0.015 (− 0.145, 0.115)	*n* = 13 0.076 (− 0.132, 0.283)
*L*_den_ school (continuous)	*n* = 1461 − 0.004 (− 0.009, 0.001)	*n* = 498 0.004 (− 0.002, 0.010)	*n* = 218 0.003 (− 0.007, 0.012)
* L*_den_ school < 50 dBA	*n* = 375 Reference	*n* = 160 Reference	*n* = 86 Reference
50–60 dBA	*n* = 921 − 0.030 (− 0.091, 0.032)	*n* = 299 − 0.008 (− 0.079, 0.063)	*n* = 124 − 0.011 (− 0.112, 0.091)
> 60 dBA	*n* = 165 − 0.113 (− 0.209, − 0.018)*	*n* = 39 0.081 (− 0.051, 0.212)	*n* = 8 0.119 (− 0.146, 0.383)

B	Regression coefficient for symptom severity (95% confidence interval)					
	Attention score (pooled)	Hyperactivity/impulsivity score (pooled)	Attention score (ADHD symptoms and diagnosis)	Hyperactivity/impulsivity score (ADHD symptoms and diagnosis)	Attention score (ADHD diagnosis)	Hyperactivity/impulsivity score (ADHD diagnosis)
*L*_den_ home (continuous)	*n* = 1,625 − 0.004 (− 0.009, 0.002)	*n* = 1,627 − 0.005 (− 0.011, − 0.000)*	*n* = 551 0.003 (− 0.004, 0.010)	*n* = 552 − 0.001 (− 0.008, 0.007)	*n* = 225 0.008 (− 0.002, 0.018)	*n* = 225 0.007 (− 0.004, 0.019
* L*_den_ home < 50 dBA	*n* = 452 Reference	*n* = 453 Reference	*n* = 170 Reference	*n* = 170 Reference	*n* = 86 Reference	*n* = 87 Reference
50–60 dBA	*n* = 1021 − 0.013 (− 0.078, 0.053)	*n* = 1022 − 0.060 (− 0.119, 0.000)	*n* = 343 0.007 (− 0.071, 0.086)	*n* = 344 − 0.043 (− 0.132, 0.046)	*n* = 125 − 0.003 (− 0.116, 0.110)	*n* = 125 0.007 (− 0.124, 0.139)
> 60 dBA	*n* = 152 − 0.079 (− 0.188, 0.030)	*n* = 152 − 0.084 (− 0.183, 0.015)	*n* = 38 − 0.048 (− 0.198, 0.101)	*n* = 38 0.007 (− 0.162, 0.177)	*n* = 13 0.088 (− 0.150, 0.326)	*n* = 13 0.066 (− 0.210, 0.343)
*L*_den_ school (continuous)	*n* = 1,459 − 0.005 (− 0.011, 0.001)	*n* = 1,461 − 0.003 (− 0.008, 0.002)	*n* = 497 0.001 (− 0.006, 0.008)	*n* = 498 0.006 (− 0.002, 0.014)	*n* = 218 − 0.001 (− 0.012, 0.010)	*n* = 218 0.006 (− 0.007, 0.018)
* L*_den_ school < 50 dBA	*n* = 375 Reference	*n* = 375 Reference	*n* = 160 Reference	*n* = 160 Reference	*n* = 86 Reference	*n* = 86 Reference
50–60 dBA	*n* = 920 − 0.021 (− 0.092, 0.051)	*n* = 921 − 0.037 (-0.103, 0.028)	*n* = 298 0.008 (− 0.073, 0.089)	*n* = 299 − 0.021 (− 0.114, 0.071)	*n* = 124 − 0.011 (− 0.126, 0.104)	*n* = 124 − 0.011 (− 0.144, 0.123)
> 60 dBA	*n* = 164 − 0.135 (− 0.247, − 0.024)*	*n* = 165 − 0.092 (− 0.193, 0.010)	*n* = 39 0.010 (− 0.140, 0.160)	*n* = 39 0.132 (− 0.039, 0.303)	*n* = 8 − 0.060 (− 0.361, 0.241	*n* = 8 0.252 (− 0.097, 0.601)

Adjusted for sex, age, parental SES, number of parents, ethnicity, perinatal circumstances and complications, and parental externalizing and internalizing problems. *N* in each cell refers to sample size. *p < 0.05

Linear regression coefficients for associations between noise and symptom severity of attentional symptoms were close to zero and not statistically significant, except for school road traffic noise > 60 dBA in the pooled sample. Children in this highest school noise category (vs. those in the < 50 dBA category) had on average − 0.135 (95% CI − 0.247, − 0.024) lower scores for attention symptom severity (Table 3b). Associations between noise and symptom severity of hyperactive/impulsive symptoms were also close to zero and not statistically significant, except for a small inverse association between residential road traffic noise and hyperactive/impulsive symptoms in the pooled sample (*β* − 0.005, 95% CI − 0.011, − 0.000; Table 3b).

### Stratification by sex, socioeconomic status, and parental psychopathology

Stratified analyses showed that in boys, residential and school road traffic noise was associated with lower risks for ADHD diagnosis (Table 4), but not with ADHD symptoms. Risk ratios for girls were generally < 1, but were not statistically significant (Table 4). Stratification by low and high parental SES revealed that the inverse association between noise and ADHD diagnosis was more evident in the high SES group, especially for school road traffic noise (Table 5). Stratification by low and high parental psychopathology revealed that the inverse association between noise and ADHD diagnosis was more evident in the high paternal internalizing problems group (Table 6).

**Table 4:** Associations between exposure to road traffic noise at home and school, and ADHD stratified by sex in the TRAILS cohort

		Risk ratio for ADHD (95% confidence interval)	
		Boys	Girls
*L*_den_ home (per 1 dBA)	ADHD symptoms	*n* = 233 1.000 (0.968, 1.032)	*n* = *108* 0.985 (0.942, 1.030)
	ADHD diagnosis	*n* = 159 0.905 (0.862, 0.950)*	*n* = 70 0.973 (0.914, 1.035)
*L*_den_ school (per 1 dBA)	ADHD symptoms	*n* = 199 0.990 (0.960, 1.020)	*n* = 94 1.014 (0.968, 1.063)
	ADHD diagnosis	*n* = 155 0.942 (0.900, 0.986)*	*n* = 67 0.939 (0.876, 1.008)
*L*_den_ home (per 1 dBA)	ADHD symptoms	*n* = 199 0.999 (0.965, 1.034)	*n* = 94 0.974 (0.929, 1.022)
*L*_den_ school (per 1 dBA)		*n* = 199 0.990 (0.961, 1.020)	*n* = 94 1.014 (0.967, 1.062)
*L*_den_ home (per 1 dBA)	ADHD diagnosis	*n* = 155 0.889 (0.844, 0.937)*	*n* = 67 0.970 (0.911, 1.034)
*L*_den_ school (per 1 dBA)		*n* = 155 0.949 (0.844, 0.937)*	*n* = 67 0.939 (0.876, 1.007)

Adjusted for age, parental SES, number of parents, ethnicity, perinatal circumstances and complications, and parental externalizing and internalizing problems. *N* in each cell refers to ADHD cases. Based on multinomial regression analysis with screen-negative for ADHD as reference group.

*p < 0.05

**Table 5:** Associations between exposure to road traffic noise at home and school, and ADHD stratified by socioeconomic status in the TRAILS cohort

		Risk ratio for ADHD (95% confidence interval)	
		Low SES	High SES
*L*_den_ home (per 1 dBA)	ADHD symptoms	*n* = 221 0.985 (0.952, 1.018)	*n* = 120 1.007 (0.967, 1.049)
	ADHD diagnosis	*n* = 116 0.924 (0.872, 0.978)*	*n* = 113 0.921 (0.871, 0.974)*
*L*_den_ school (per 1 dBA)	ADHD symptoms	*n* = 188 0.992 (0.960, 1.025)	*n* = 105 1.008 (0.970, 1.048)
	ADHD diagnosis	*n* = 113 0.985 (0.935, 1.037)	*n* = 109 0.909 (0.859, 0.961)*
*L*_den_ home (per 1 dBA)	ADHD symptoms	*n* = 188 0.981 (0.946, 1.017)	*n* = 105 1.006 (0.962, 1.051)
*L*_den_ school (per 1 dBA)		*n* = 188 0.992 (0.960, 1.023)	*n* = 105 1.008 (0.970, 1.048)
*L*_den_ home (per 1 dBA)	ADHD diagnosis	*n* = 113 0.924 (0.870, 0.981)*	*n* = 109 0.907 (0.855, 0.963)*
*L*_den_ school (per 1 dBA)		*n* = 113 0.985 (0.935, 1.038)	*n* = 109 0.918 (0.855, 0.963)*

Adjusted for age, parental SES, number of parents, ethnicity, perinatal circumstances and complications, and parental externalizing and internalizing problems. *N* in each cell refers to ADHD cases. Based on multinomial regression analysis with screen-negative for ADHD as reference group

*p < 0.05

**Table 6:** Associations between exposure to road traffic noise at home and school, and ADHD stratified by parental psychopathology in the TRAILS cohort

		Risk ratio for ADHD (95% confidence interval)			
		Low paternal internalizing problems	High paternal internalizing problems	Low paternal externalizing problems	High paternal externalizing problems
*L*_den_ home (per 1 dBA)	ADHD symptoms	*n* = 167 1.015 (0.979, 1.052)	*n* = 174 0.970 (0.935, 1.007)	*n* = 272 0.988 (0.960, 1.016)	*n* = 69 1.009 (0.951, 1.071)
	ADHD diagnosis	*n* = 81 0.947 (0.886, 1.012)	*n* = 148 0.919 (0.875, 0.965)*	*n* = 168 0.926 (0.887, 0.967)*	*n* = 61 0.934 (0.851, 1.024)
*L*_den_ school (per 1 dBA)	ADHD symptoms	*n* = 140 1.004 (0.969, 1.042)	*n* = 153 0.996 (0.962, 1.031)	*n* = 232 0.994 (0.966, 1.023)	*n* = 61 1.014 (0.961, 1.069)
	ADHD diagnosis	*n* = 77 0.947 (0.889, 1.009)	*n* = 145 0.949 (0.906, 0.994)*	*n* = 164 0.937 (0.896, 0.979)*	*n* = 58 0.974 (0.902, 1.051)
*L*_den_ home (per 1 dBA)	ADHD symptoms	*n* = 140 1.013 (0.974, 1.054)	*n* = 153 0.966 (0.928, 1.005)	*n* = 232 0.982 (0.952, 1.013)	*n* = 61 1.018 (0.955, 1.084)
*L*_den_ school (per 1 dBA)		*n* = 140 1.004 (0.968, 1.041)	*n* = 153 0.996 (0.962, 1.031)	*n* = 232 0.995 (0.967, 1.023)	*n* = 61 1.015 (0.962, 1.071)
*L*_den_ home (per 1 dBA)	ADHD diagnosis	*n* = 77 0.938 (0.874, 1.007)	*n* = 145 0.911 (0.866, 0.959)*	*n* = 164 0.921 (0.880, 0.964)	*n* = 58 0.924 (0.838, 1.019)
*L*_den_ school (per 1 dBA)		*n* = 77 0.951 (0.893, 1.012)	*n* = 145 0.952 (0.908, 0.997)*	*n* = 164 0.944 (0.904, 0.986)	*n* = 58 0.969 (0.896, 1.048)

Note: adjusted for age, parental SES, number of parents, ethnicity, perinatal circumstances and complications, and parental externalizing and internalizing problems. *N* in each cell refers to ADHD cases. Based on multinomial regression analysis with screen-negative for ADHD as reference group

*p < 0.05

### Sensitivity analysis

Sensitivity analyses with a stricter criterion (95th percentile cut-off vs. 90th percentile cut-off) for ADHD symptoms did not result in different results: higher noise levels were associated with lower risks for a clinical ADHD diagnosis, but not ADHD symptoms. Although risk ratios for ADHD symptoms in relation to residential road traffic noise became > 1, these were not statistically significant (Table 7). Additional adjustment for pregnancy duration and birth weight, and household crowding did not change the results (data not shown). Stratified analysis for those with low and high noise sensitivity did not reveal a differential association between road traffic noise and ADHD symptoms (Table 7). Associations between noise sensitivity itself and ADHD symptoms were not statistically significant, but results tended to show higher odds for ADHD symptoms with increasing noise sensitivity (Table 8).

**Table 7:** Associations between exposure to road traffic noise at home and school, and ADHD stratified by noise sensitivity in the TRAILS population cohort

		Odds ratio for ADHD (95% confidence interval)	
		Low noise sensitivity	High noise sensitivity
*L*_den_ home (per 1 dBA)	ADHD symptoms	*n* = 1051 0.991 (0.960, 1.023)	*n* = 312 1.004 (0.953, 1.058)
*L*_den_ school (per 1 dBA)	ADHD symptoms	*n* = 946 1.001 (0.972, 1.032)	*n* = 275 1.021 (0.969, 1.075)
*L*_den_ home (per 1 dBA)	ADHD symptoms	*n* = 946 0.985 (0.952, 1.019)	*n* = 275 1.005 (0.947, 1.068)
*L*_den_ school (per 1 dBA)		*n* = 946 1.002 (0.972, 1.032)	*n* = 275 1.021 (0.969, 1.076)

Adjusted for age, parental SES, number of parents, ethnicity, perinatal circumstances and complications, and parental externalizing and internalizing problems. *N* in each cell refers to ADHD cases. Based on logistic regression analysis with screen-negative for ADHD as reference group

**Table 8:** Associations between noise sensitivity and ADHD in the TRAILS population cohort

	*N* cases	Odds ratio for ADHD (95% confidence interval)			
		m1	m2	m3	m4
Noise sensitivity					
Almost never true	79	Reference	Reference	Reference	Reference
Usually not true	44	0.897 (0.593, 1.356)	0.944 (0.619, 1.441)	0.909 (0.592, 1.396)	0.901 (0.586, 1.386)
Sometimes true	111	1.181 (0.851, 1.639)	1.243 (0.890, 1.737)	1.226 (0.874, 1.721)	1.233 (0.878, 1.732)
Usually true	39	1.072 (0.693, 1.658)	1.166 (0.745, 1.823)	1.143 (0.726, 1.798)	1.170 (0.742, 1.843)
Almost always true	39	1.406 (0.900, 2.196)	1.306 (0.829, 2.058)	1.226 (0.771, 1.951)	1.256 (0.788, 2.001)

*M1* adjusted for sex, age; *M2* M1 + parental SES, number of parents, ethnicity; *M3* M2 + perinatal circumstances and complications; *M4* M3 + parental externalizing and internalizing problems

Finally, additional adjustment for residential environment quality in the population sample did not change the results (Supplemental Material, Table S2).

## Discussion

In this cross-sectional study of 1710 children, we found no evidence for a harmful association between road traffic noise and ADHD. Higher noise levels at the residence and school were associated with a lower risk of a clinical ADHD diagnosis, but not with ADHD symptoms in the population cohort or with symptom severity. The results were consistent after adjustment for various known risk factors for ADHD and socioeconomic status and for different cut-offs of ADHD symptoms.

Our results differ from the previous studies that did observe harmful associations between road traffic noise and ADHD symptoms [7–10]. Average noise levels in these previous studies were generally higher than in our study, and these previous studies focused solely on ADHD symptoms and did not assess clinical diagnosis [7–10], potentially explaining the different findings. Furthermore, study populations in these previous studies were younger than our sample, suggesting that road traffic noise exposure at younger ages might be more harmful [7–10]. In line with the results in our study, road traffic noise annoyance (reported by the parent) was not associated with more hyperactivity symptoms in a German study of 1185 children [33]. However, since that study used parental noise annoyance as a proxy for children’s noise exposure, it is less comparable to our study.

Given the results of previous studies [7–10] and the absence of a plausible mechanism for a protective association between traffic noise and ADHD, we consider it unlikely that our results represent a true protective association between traffic noise and ADHD. This protective association was not observed in the population cohort and neither did we observe a clear association between noise and symptom severity. This suggests that the observed protective association might be a chance finding. The children with a clinical ADHD diagnosis are different than those who screened positive for ADHD symptoms in the population cohort. The group with an ADHD diagnosis was selected based on referral to an outpatient clinic, while the children who screened positive for enhanced ADHD symptom levels were selected from the general population and their problems were not necessarily severe enough to warrant a clinical diagnosis. Those with a clinical ADHD diagnosis might also reflect a group that has access to the healthcare system and selection bias might be present. The children with ADHD diagnoses also differed from children that screened positive for ADHD symptoms in terms of a higher parental socioeconomic status, a lower frequency of a foreign-born parent, and a higher frequency of complications during pregnancy or delivery. While inclusion of these covariates did not alter the results, other unmeasured factors different in the clinical cohort than in the population cohort might have led to residual confounding. Failing to adjust for factors related to low noise levels and high ADHD diagnoses could be an explanation for the unexpected inverse association between noise and ADHD. The inverse relationship between noise and ADHD diagnosis was more apparent in the higher SES group and in those with parents with internalizing problems, potentially indicating selection bias or residual confounding. As hypothesized previously [34], parental psychopathology could have resulted in residential self-selection into areas with low noise exposure, potentially explaining our findings. Similar to our study, a previous study from Spain found an unexpected association between ambient air pollution level and higher ADHD prevalence. The authors hypothesized that parental mental health could have resulted in residential self-selection, assuming that parents with the existing ADHD symptoms and their predisposed children would have moved to more quiet areas with less air pollution, and in our case, less noise exposure. We were not able to confirm this hypothesis, as no residential history of the parents or family was available. This underlines the importance of longitudinal studies with a life-course approach, where long-term processes are studied that link non-communicable disease to exposures throughout life, starting from the preconception period [35]. The observation that the inverse association between residential and school road traffic noise was associated with lower risks for ADHD diagnosis in boys, but not girls, might be explained by the higher prevalence of ADHD in boys compared to girls.

Finally, the association between road traffic noise and ADHD did not differ by low or high noise sensitive children. This was assessed in the population sample only, and potential effect modification could thus not be studied in the clinical sample. Noise sensitivity refers to the variability in reactivity to different sources of noise in individuals. Although not studied extensively in children, noise sensitivity may be an important factor in the association between noise exposure, annoyance, and behavioral problems [23, 36, 37]. Although not statistically significant that might be due to limited statistical power, our results showed some indication for an association between noise sensitivity and ADHD symptoms. This is generally in line with the previous studies and hypotheses [23, 36, 37]. Future studies should consider noise sensitivity in children when studying the association between noise and ADHD as it will shed light on vulnerable groups and potential mechanisms.

## Strengths and limitations

This study was based on a rich data set that enabled us to adjust for potential confounders and perform a number of sensitivity analyses. Children spend a large part of their time at school, and thus, having road traffic noise data for both homes and schools was a major advantage. Limitations include that the period of cohort’s measurements and the year of the noise model are 10 years apart, and may have led to exposure misclassification. Potential exposure misclassification is assumed to be small, assuming that spatial variability in noise exposure remained similar, and that traffic intensity would not have strongly increased. These assumptions are reasonable, because historical changes in infrastructure (e.g., construction of new major roads), and thus in spatial variability, are assumed to be limited, especially in the urban areas of our study area where the largest part of our study population lives [38]. Note further that even a doubling of traffic intensity would result in an increase of 3 dB(A) in noise exposure, which is barely audible. Considering that a doubling of traffic intensity is a large increase, and not very likely over this 10-year period, we assumed that absolute noise levels would not have changed significantly [39]. We had no data on air pollution exposure, while this is highly correlated with traffic noise and is potentially a risk factor for ADHD symptoms and cognitive impairment [40–43], although not consistently [34]. Factors that could moderate or potentially confound the association between noise and ADHD are characteristics of the home and school (e.g., crowding, bedroom, and classroom orientation). Crowding in the household and residential environment quality did not seem to play a role, but for the other factors, this is unclear, since they were not assessed in this study. Such unmeasured home or school environment factors could differ between low and high traffic noise areas, and could be associated with ADHD prevalence, and may have biased our results. Future studies should take these factors into account. Although the TRAILS cohort is longitudinal, our analysis was cross-sectional and only used data from the first measurement wave. We, therefore, cannot determine the direction of the observed associations and future research should assess the longitudinal association between road traffic noise and ADHD. Finally, in some cases, sample size was low and those estimates should be interpreted with caution.

## Conclusion

This cross-sectional study found no evidence for a harmful association between road traffic noise and ADHD. Longitudinal studies should focus on the association between traffic noise and ADHD in schools and at home.

## Electronic supplementary material

Below is the link to the electronic supplementary material.

Supplementary file1 (DOCX 15 kb)
