# Supplementary material for: Associations between road traffic noise exposure at home and school and ADHD in school-aged children: the TRAILS study
Source: Eur Child Adolesc Psychiatry. 2020 Apr 3;30(1):155–67. doi: 10.1007/s00787-020-01521-8 (PMC7864841; doi:10.1007/s00787-020-01521-8)
Supplement: Supplementary file 1 — Supplementary file1 (DOCX 15 kb) [file 787_2020_1521_MOESM1_ESM.docx]

Supplemental Tables

Table S1. Associations between exposure to road traffic noise at home (n=1710) and school (n=1538), and ADHD in the TRAILS cohort (Sensitivity analysis - with alternative cut off for ADHD symptoms)

|  |  |  | Risk ratio for ADHD (95% confidence interval) | | | |
| --- | --- | --- | --- | --- | --- | --- |
|  |  | N cases | m1 | m2 | m3 | m4 |
| L_den_ home (per 1 dBA) | ADHD symptoms | 126 | 1.007 (0.971, 1.044) | 1.008 (0.972, 1.046) | 1.009 (0.972, 1.048) | 1.009 (0.972, 1.047) |
|  | ADHD diagnosis | 229 | 0.943 (0.915, 0.972)* | 0.943 (0.915, 0.972)* | 0.932 (0.897, 0.968)* | 0.931 (0.896, 0.967)* |
|  |  |  |  |  |  |  |
| L_den_ school (per 1 dBA) | ADHD symptoms | 110 | 0.977 (0.942, 1.014) | 0.984 (0.948, 1.021) | 0.984 (0.948, 1.022) | 0.986 (0.949, 1.023) |
|  | ADHD diagnosis | 222 | 0.940 (0.914, 0.968)* | 0.944 (0.917, 0.972)* | 0.943 (0.909, 0.978)* | 0.944 (0.910, 0.980)* |
|  |  |  |  |  |  |  |
| L_den_ home (per 1 dBA) | ADHD symptoms | 110 | 1.006 (0.968, 1.046) | 1.008 (0.969, 1.048) | 1.008 (0.969, 1.049) | 1.008 (0.969, 1.049) |
| L_den_ school (per 1 dBA) |  |  | 0.977 (0.942, 1.014) | 0.984 (0.948, 1.022) | 0.985 (0.948, 1.022) | 0.986 (0.950, 1.024) |
| L_den_ home (per 1 dBA) | ADHD diagnosis | 222 | 0.944 (0.915, 0.973)* | 0.943 (0.915, 0.973)* | 0.927 (0.891, 0.965)* | 0.925 (0.888, 0.962)* |
| L_den_ school (per 1 dBA) |  |  | 0.941 (0.915, 0.969)* | 0.945 (0.919, 0.973)* | 0.946 (0.912, 0.981)* | 0.947 (0.913, 0.983)* |

Note: M1: adjusted for sex, age; M2: M1+ parental SES, number of parents, ethnicity; M3: M2+ perinatal circumstances and complications; M4: M3+ parental externalizing and internalizing problems. Based on multinomial regression analysis with screen-negative for ADHD as reference group (n=1355/1206). *p<.05.

Table S2. Associations between exposure to road traffic noise at home (n=1386) and school (n=1223), and ADHD in the TRAILS population cohort (Sensitivity analysis - with additional adjustment for quality of the residential environment)

|  |  |  | Odds ratio for ADHD (95% confidence interval) |
| --- | --- | --- | --- |
|  |  | N cases |  |
| L_den_ home (per 1 dBA) | ADHD symptoms | 333 | 0.993 (0.965, 1.021) |
|  |  |  |  |
| L_den_ school (per 1 dBA) | ADHD symptoms | 288 | 0.997 (0.971, 1.023) |
|  |  |  |  |
| L_den_ home (per 1 dBA) | ADHD symptoms | 288 | 1.000 (0.961, 1.020) |
| L_den_ school (per 1 dBA) |  |  | 1.000 (0.971, 1.023) |

Note: adjusted for sex, age, parental SES, number of parents, ethnicity, perinatal circumstances and complications, parental externalizing and internalizing problems, and quality of the residential environment. Based on logistic regression analysis with screen-negative for ADHD as reference group.
